# Supplementary material for: Heterodimerization of staphylococcal phage φ2638A endolysin isoforms and their functional role in bacterial lysis
Source: Microlife. 2025 Jun 10;6:uqaf011. doi: 10.1093/femsml/uqaf011 (PMC12203907; doi:10.1093/femsml/uqaf011)
Supplement: uqaf011_Supplemental_File [file uqaf011_supplemental_file.pdf]

HETERODIMERIZATION OF STAPHYLOCOCCAL PHAGE Φ2638A ENDOLYSIN ISOFORMS AND THEIR FUNCTIONAL ROLE IN BACTERIAL LYSIS

SUPPLEMENTARY INFORMATION

SUPPLEMENTARY TABLE S1. Oligonucleotides used for the amplification of genomic DNA fragments required for synthetic assembly of bacteriophage genomes.

Fragment	Name	Primer sequence (5' → 3')	Phage	Template
f1	419 (F)	ACTATCGGAACGTCCAGATTTAGC	φ2638A *ply*$_{WT-HA}$	φ2638A WT
	412 (R)	TACCGACTGCGCGCATCTGAC		
f2.1	413 (F)	TCTATCCGGAATGTAGCAGGTCAG		
	HA_(R)	TATTTTAAGCGTAATCTGGAACATCGTATGGGTATTTAATTTCGCCCCACAACTTACCAACTTTAC		
f3.1	HA_(F)	ATTAAATACCCATACGATGTTCCAGATTACGCTTAAAATATGATATACTATGTATATCCACGACATGATTAG		
	416 (R)	CTTGATAGCCCAATGCCAATTCTG		
f4	417 (F)	CAAAAAGGTAAAGTATCAGAATTGGCATTG		
	420 (R)	ATTGTTCAGGTCATACGCTAAATCTG		
f2.2	413 (F)	TCTATCCGGAATGTAGCAGGTCAG	φ2638A *ply*$_{FL}$	φ2638A WT
	CTC_(R)	GTTTGAATAGATATGTTTGAGCTCTTTCACGCTCCC		
f3.2	CTC_(F)	GAATGGGAGCGTGAAAGAGCTCAAACATATCTATTC		
	416 (R)	CTTGATAGCCCAATGCCAATTCTG		
f3.3	CTC_(F)	GAATGGGAGCGTGAAAGAGCTCAAACATATCTATTC	φ2638A *ply*$_{FL-HA}$	φ2638A *ply-ha*
	416 (R)	CTTGATAGCCCAATGCCAATTCTG		
f2.4	413 (F)	TCTATCCGGAATGTAGCAGGTCAG	φ2638A *ply*$_{SV}$	φ2638A WT
	Δ_M23_(R)	TTTGAATAGATATGTTTCATGTCACTTCAGCCCTTTCTCTTTAAGGTATTCC		
f3.4	Δ_M23_(F)	AGAAAGGGCTGAAGTGACATGAAACATATCTATTCAAACCATATTAAAGG		
	416 (R)	CTTGATAGCCCAATGCCAATTCTG		
f2.5	413 (F)	TCTATCCGGAATGTAGCAGGTCAG	φ2638A *ply*$_{SV-HA}$	φ2638A *ply*$_{WT-HA}$
	Δ_M23_(R)	TTTGAATAGATATGTTTCATGTCACTTCAGCCCTTTCTCTTTAAGGTATTCC		
f3.5	Δ_M23_(F)	AGAAAGGGCTGAAGTGACATGAAACATATCTATTCAAACCATATTAAAGG		
	416 (R)	CTTGATAGCCCAATGCCAATTCTG		

SUPPLEMENTARY TABLE S2. Oligonucleotides and final protein expression plasmids

Primer name and sequence (5'→3')	Template	Construct	Vector	6×His[1)]
NdeI_Ply2638A_F<br>GATCCATATGCTAACTGCTATTGAC<br>Ply2638 _BamHI_R<br>TAATGGATCCTTATTTAATTTCGCCC	Ply2638A 1–486	Ply_{WT}	pET302	-
NdeI_Ply2638A_F<br>GATCCATATGCTAACTGCTATTGAC<br>2638a_CTC_180_mut_F<br>GTGAAAGAGCTCAAACATATCTATTC<br>2638a_CTC_180_mut_R<br>GATATGTTTGAGCTCTTTCACGCTCC<br>Ply2638 _BamHI_R<br>TAATGGATCCTTATTTAATTTCGCCC	Ply_{WT}	Ply_{FL}	pET302	-
Ply2638_short_NdeI_F<br>TCACCATATGAAACATATCTATTCAAACC<br>Ply2638 _BamHI_R<br>TAATGGATCCTTATTTAATTTCGCCC	Ply_{WT}	Ply_{SV}	pET302	-
M23-2638_XhoI_F<br>ATAGCTCGAGATGCTGACC<br>M23-2638_BamHI_R<br>ATCGGGATCCTTAGTTTTTGC	Synthetic DNA[4)]	M23	pET302	NT
Ami(GA)_NdeI_F<br>TAATCACATATGGGTAGCGTGAAAGAGC<br>Ami(GA)_BamHI_R<br>AATTGGATCCTTAACCGTCGTAGTAATG<br>TTTG	Synthetic DNA[4)]	Ami	pET302	-
CBD2638_NdeI_F<br>ATAATACATATGTGGAAACAGAACAAAG<br>ATGGC<br>CBD(GA2)_BamHI_R<br>ATTAGGATCCTTATTTGATTTCACCCCA<br>CAG	Synthetic DNA[4)]	CBD	pET200	-
-	-	HGFP[2)]	pQE30	NT
-	-	HGFP_SH3b2638A[3)]	pQE30	NT
-	-	HGFP_SH3bLST[3)]	pQE30	NT

[1)] Presence of the 6×Histidine tag: '-' no tag; 'NT' N-terminal His-tag

[2)] Loessner et al. (2002)

[3)] Doctoral Thesis, Fritz Eichenseher 2011, ETH Zurich

[4)] GeneArt (ThermoFisher)

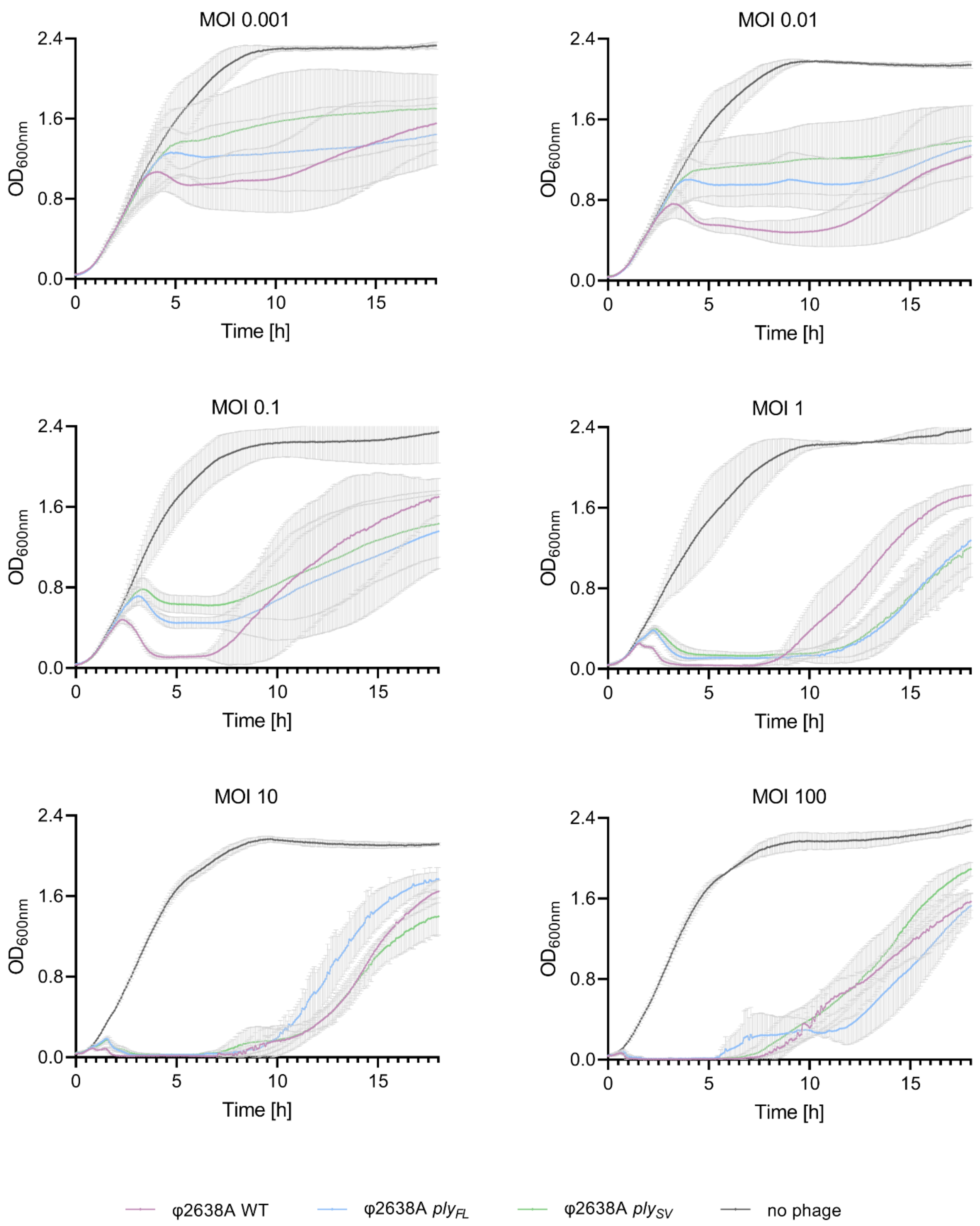

SUPPLEMENTARY FIGURE S1. **Turbidity reduction assays (TRAs) of wildtype and engineered φ2638A phages at different MOIs.** Wildtype φ2638A (purple), φ2638A*plyFL* (blue), φ2638A*plySV* (green), or buffer alone as control (black) were added to 10^8 CFU/mL *S. pseudointermedius* 2854 cells at different MOIs with bacteriolytic activity measured over 18 hours via optical density at OD_{600nm}. MOIs ranged from 0.001 to 100, corresponding to 10^5 to 10^{10} PFU/mL of phages. The initial 8 hours of infection for the MOI 0.1 (10^7 PFU/mL) data are presented in FIGURE 1C.

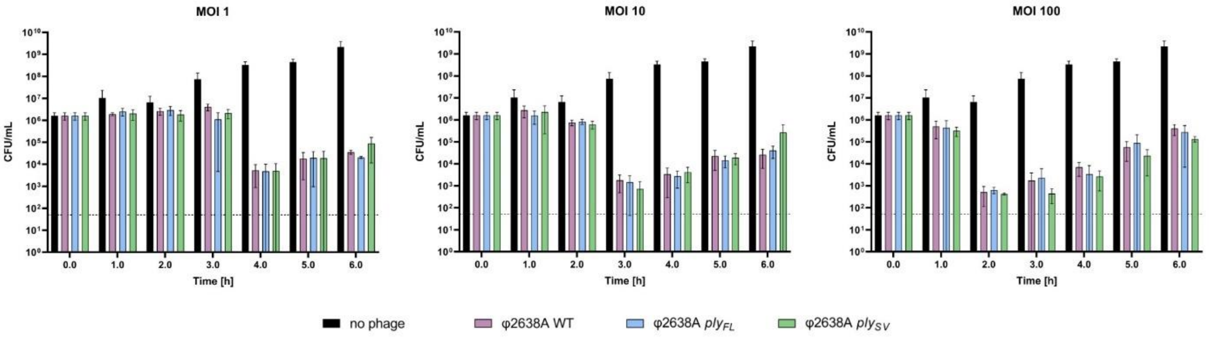

SUPPLEMENTARY FIGURE S2. **Time-kill assays (TKAs) of wildtype and engineered φ2638A phages at different MOIs.** Wildtype and engineered phages were added to 4 x 10^7 CFU/mL *S. pseudointermedius* 2854 cells at different MOIs with bacteriolytic activity measured for six hours. Samples were taken, serially diluted, and plated on agar plates every hour. Surviving colonies were quantified after 16-hour incubation at 37 °C. MOIs ranged from 1.0 to 100, corresponding to 10^7 to 10^{10} PFU/mL of phages. Dashed line represents detection limit.

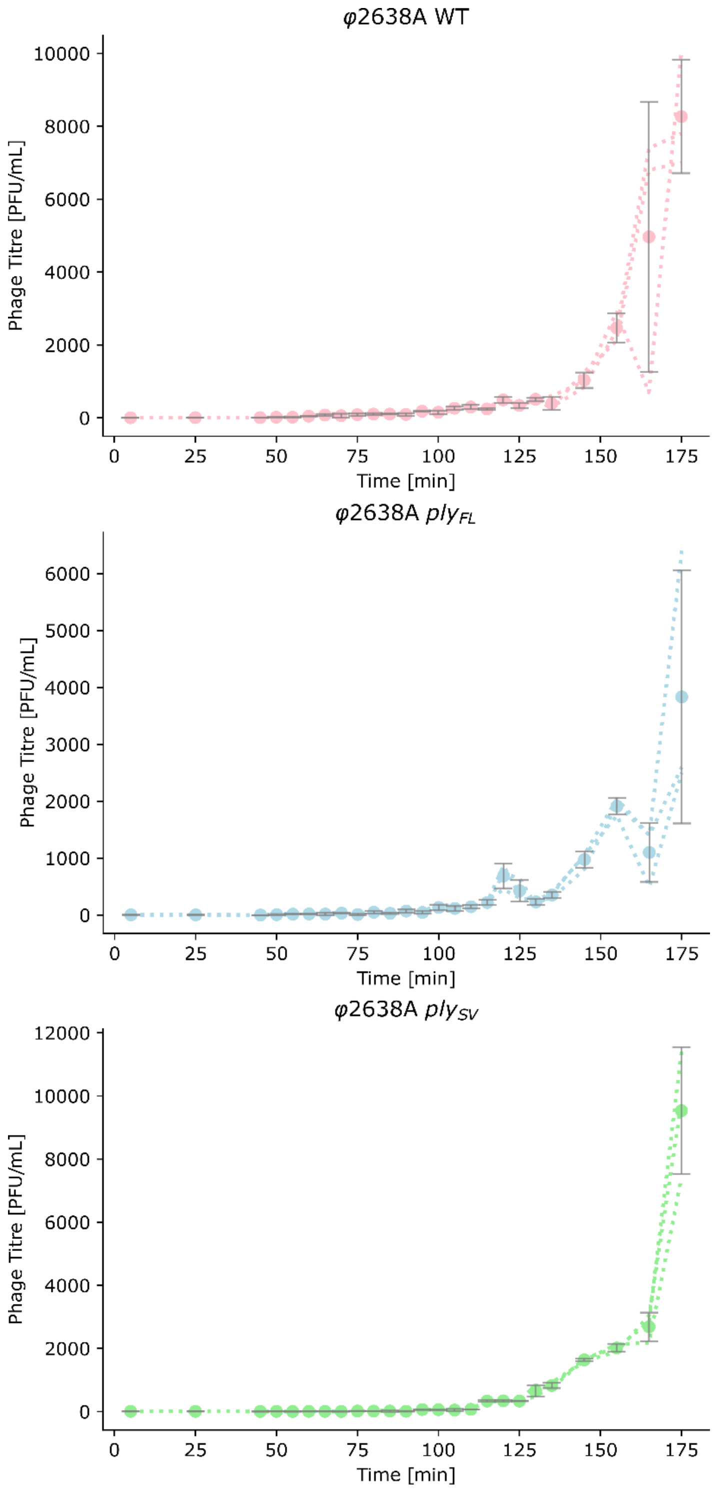

SUPPLEMENTARY FIGURE S3. **One-step growth curves of wildtype and engineered φ2638A phages**. Wildtype and engineered phages were added to *S. pseudointermedius* 2854 cells and allowed to adsorb for 5 min before removing the unbound phage in the supernatant. The exposed cells where then plated at defined intervals over 180 min to measure phage titers. A detailed analysis of the initial phage production (first 110 min) was performed and is shown in FIGURE 1D. Error bars represent the mean ± standard deviation of the technical replicates with the dotted lines showing the individual replicates.

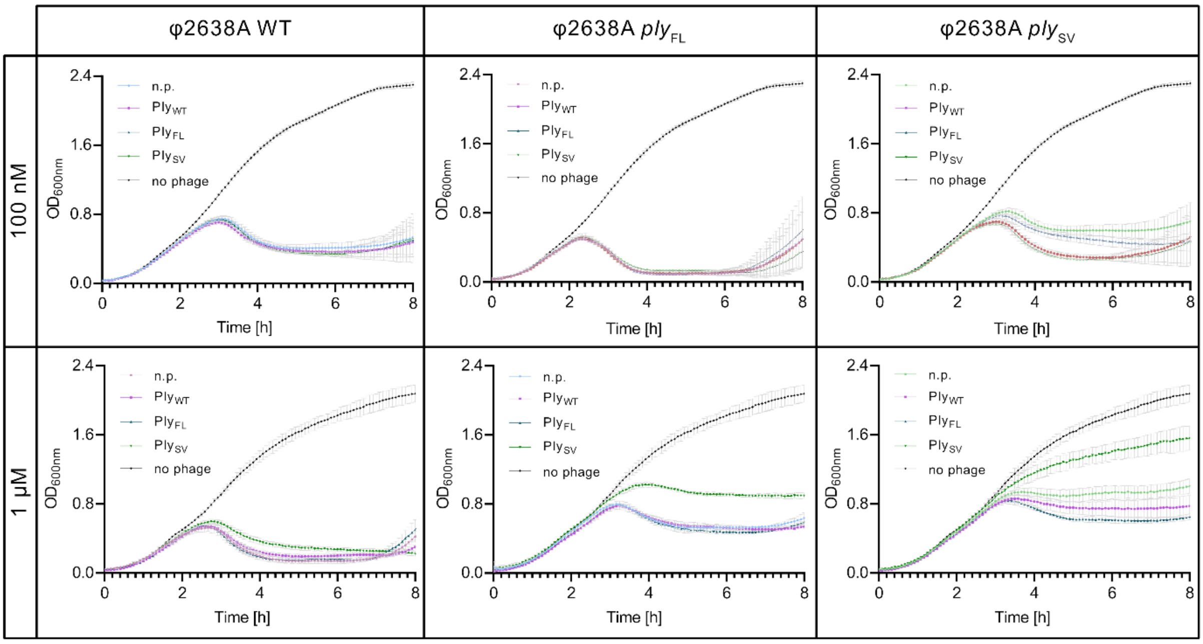

SUPPLEMENTARY FIGURE S4. **Turbidity reduction assays (TRAs) of wildtype and engineered φ2638A phages complemented with high amounts of recombinant endolysin**. Wildtype φ2638A and engineered phages, φ2638A*plyFL* and φ2638A*plySV* were added to 10^8 CFU/mL *S. pseudointermedius* 2854 cells at an MOI of 0.1 (10^7 PFU/mL) and complemented with 100 nM or 1 µM of recombinant PlyWT (the native mix of isoforms), PlyFL, or PlySV, or no protein (n.p.) as control. Each experiment contains an uninfected bacterial culture (no phage) as negative control. Experiments with a 100 nM protein supplemented were performed in biological triplicates with technical triplicates. For TRAs with 1 µM protein supplemented only technical replicates were performed. Error bars represent the mean ± standard deviation.

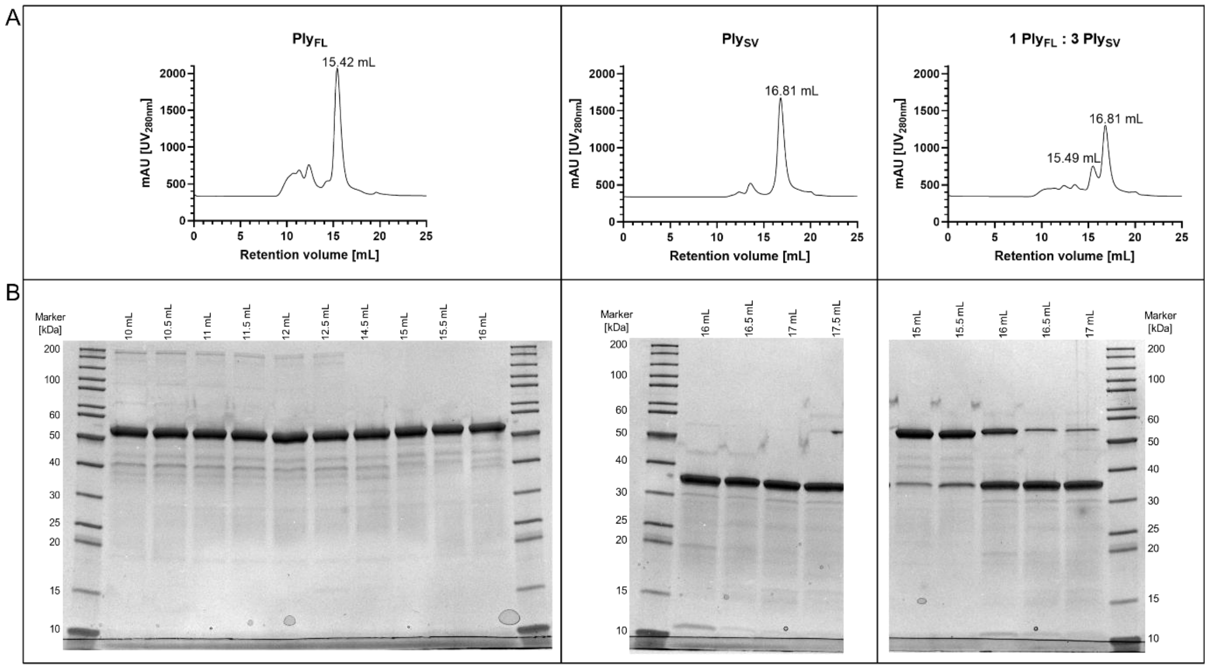

SUPPLEMENTARY FIGURE S5. **Size exclusion chromatography (SEC) of Ply_{FL}, Ply_{SV}, and a 1:3 ratio thereof with corresponding SDS-PAGE gel of selected fractions. (A)** Analytical SEC was performed with protein concentrations of 40 µM collecting 0.5 mL fractions. **(B)** Selected fractions corresponding to the observed peaks run on an SDS-PAGE gel.

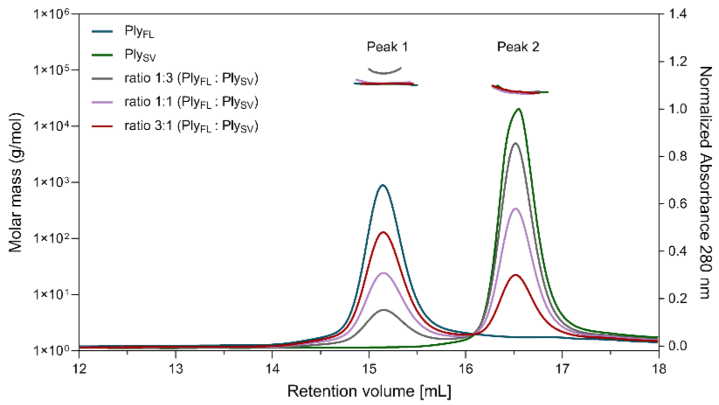

Sample	Peak 1	Peak 2
Ply_{FL} (55.5 kDa)	56.4 (± 0.6)	-
Ply_{SV} (35.3 kDa)	-	40.8 (± 0.3)
Ply_{FL} : Ply_{SV} (1:3)	97.0 (± 1.2)	42.6 (± 0.4)
Ply_{FL} : Ply_{SV} (1:1)	57.4 (± 0.5)	42.5 (± 0.9)
Ply_{FL} : Ply_{SV} (3:1)	59.1 (± 0.7)	39.7 (± 0.4)

SUPPLEMENTARY FIGURE S6. **Size exclusion chromatography with multi-angle static light scattering (SEC-MALS) analysis of Ply2638A isoform ratios.** The oligomeric states of Ply_{FL}, Ply_{SV}, and combinations of different ratios, 1:1, 3:1, 1:3 (w/w) were determined by SEC-MALS at a concentration of 1 mg/mL. The curves represent UV absorption at 280 nm, with the calculated molar mass (*y*-axis) for the respective peaks indicated above the corresponding peaks and summarized in the table on the right. BSA (bovine serum albumin) served as a reference standard during calibration.

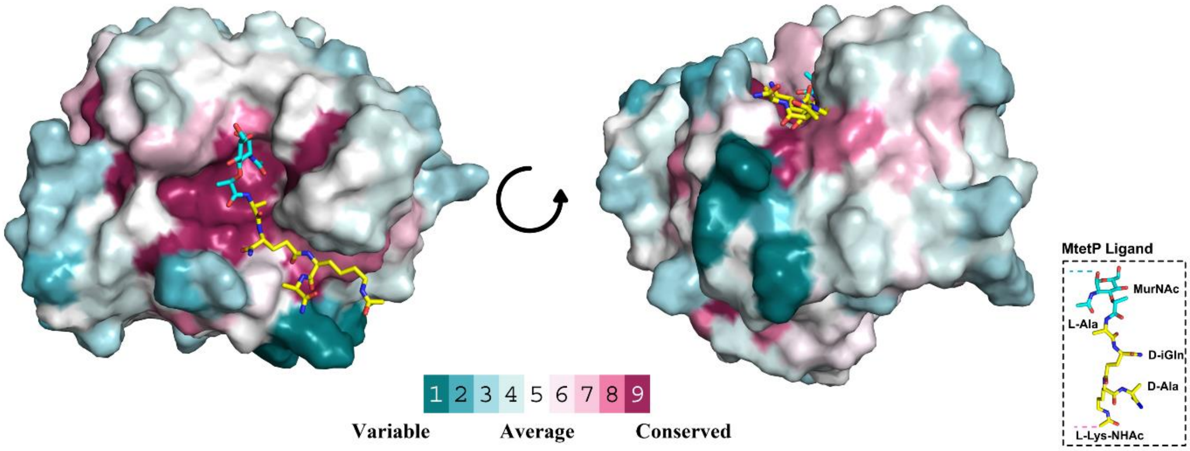

SUPPLEMENTARY FIGURE S7. **Amino acid conservation of the Ply2638A amidase domain.** Analysis was performed using the ConSurf server (Ashkenazy et al. 2016) with the default parameters for homolog search and multiple sequence alignment. Residues are colored according to the ConSurf conservation score (see legend). The muramyltetrapeptide (MtetP) ligand representative of *S. aureus* peptidoglycan was modelled into the active site by superpositioning with the MtetP co-crystallized structure of *S. aureus* autolysin, AmiA (PDB ID: 4KNL; Z-score 18.2; RMSD 2.3 Å) (Büttner et al. 2014).

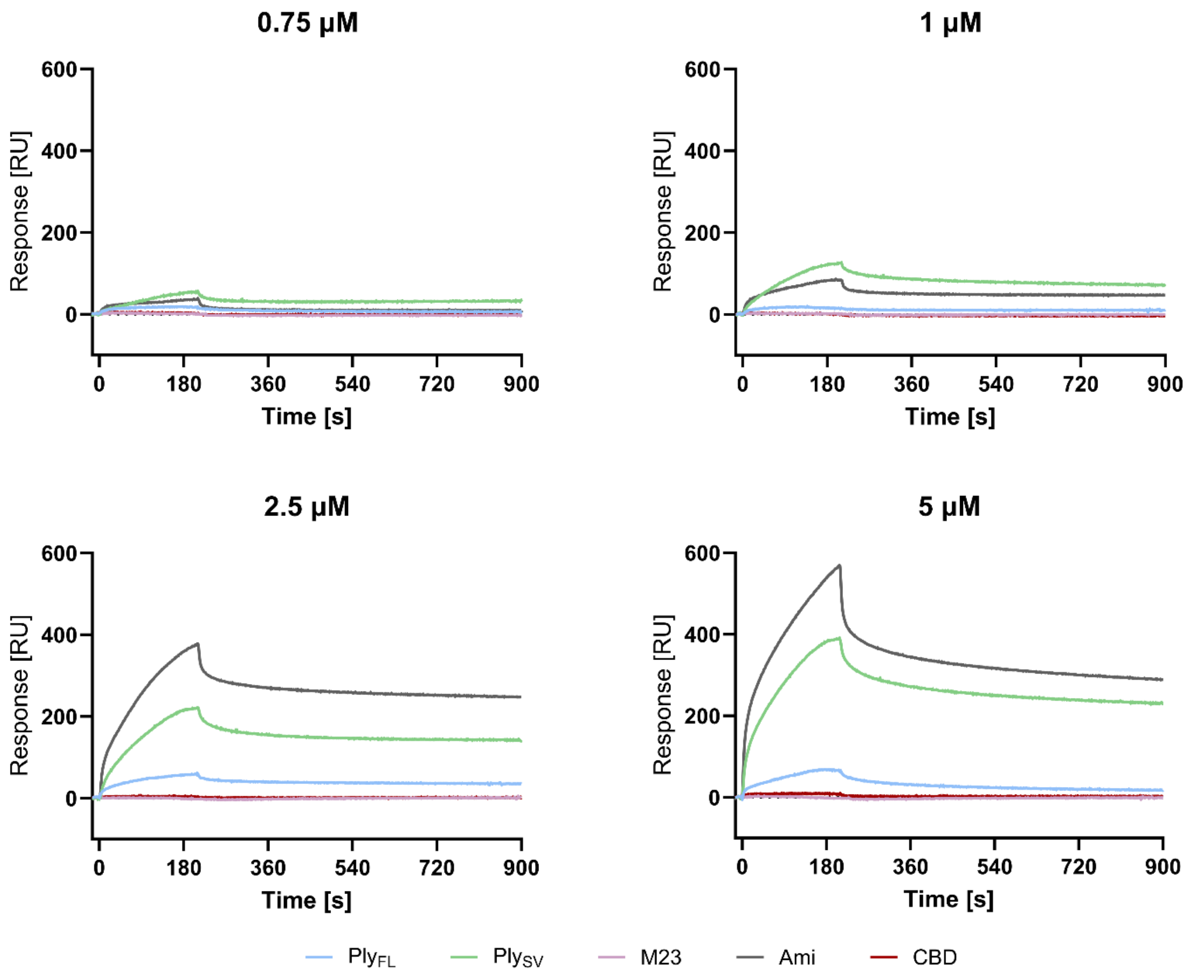

SUPPLEMENTARY FIGURE S8. **SPR data indicating interactions between amidase-containing constructs.** SPR sensorgrams of the analytes Ply$_{FL}$ (light blue), Ply$_{SV}$ (light green) and single domains M23 (purple), Ami (dark grey) and CBD (red) interacting with the ligand Ply$_{FL}$ immobilized on the chip surface. Four different analyte concentration were tested for all five constructs: 0.75 µM, 1 µM, 2.5 µM (shown in FIGURE 2E), and 5 µM.

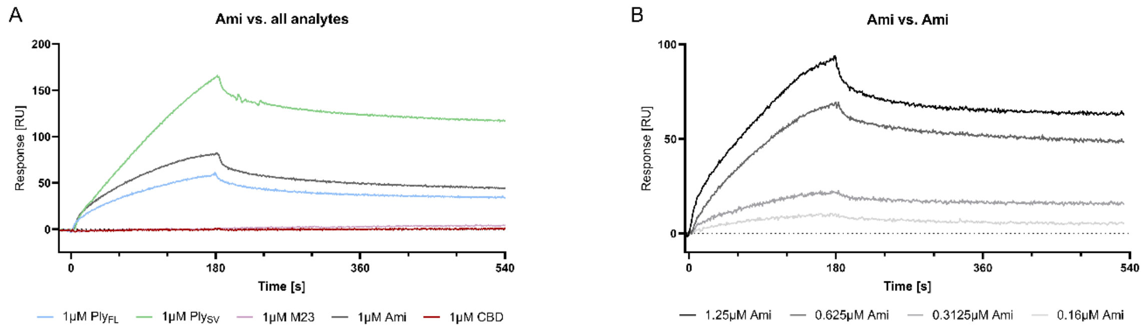

SUPPLEMENTARY FIGURE S9. **SPR data indicating interactions between two amidases.** A) SPR sensorgrams of the analytes Ply$_{FL}$ (light blue), Ply$_{SV}$ (light green) and single domains M23 (purple), Ami (dark grey) and CBD (red) interacting with the ligand Ami immobilized on the chip surface at 1 µM analyte concentration. B) Four different Ami concentration were tested against immobilized Ami: 1.25 µM, 0.625 µM, 0.3125 µM, and 0.16 µM.
